# Transcriptomic skin niches in systemic sclerosis underpin a role for mitochondrial dysfunction

**DOI:** 10.1093/bjd/ljaf119

**Published:** 2025-04-02

**Authors:** Junqin Wang, Dylan Hennessey, Aishwarya Iyer, Sandra O’Keefe, Lamia Khan, Desiree Redmond, Mohammed Osman, Robert Gniadecki

**Affiliations:** Division of Dermatology, University of Alberta, Edmonton, AB, Canada; Division of Dermatology, University of Alberta, Edmonton, AB, Canada; Division of Dermatology, University of Alberta, Edmonton, AB, Canada; Division of Dermatology, University of Alberta, Edmonton, AB, Canada; Division of Rheumatology, University of Alberta, Edmonton, AB, Canada; Division of Rheumatology, University of Alberta, Edmonton, AB, Canada; Division of Rheumatology, University of Alberta, Edmonton, AB, Canada; Division of Dermatology, University of Alberta, Edmonton, AB, Canada

## Abstract

Systemic sclerosis (SSc) is a chronic inflammatory disease characterized by fibrosis, vasculopathy and immune dysregulation. Using spatial transcriptomics on the dermis of 11 patients with SSc, we identified distinct transcriptomic niches associated with fibrosis, immune activation and mitochondrial dysfunction. Our findings highlight mitochondrial dysfunction as a central mechanism in SSc pathogenesis, ranging from adaptive remodelling in immune zones to metabolic collapse in fibrotic zones, underscoring mitochondria as a therapeutic target.

Dear Editor, Systemic sclerosis (SSc), or scleroderma, is a chronic, multiorgan inflammatory disease characterized by vasculopathy, fibrosis of the skin and visceral organs, and immune dysregulation. As the pathogenesis of SSc remains elusive, there have been only modest clinical improvements from treatments targeting fibrosis and/or inflammatory responses.^1^

Based on our previous studies analysing spatial patterning of inflammation in the skin,^2^ we hypothesized that the pathogenic processes in SSc are not diffusely uniform throughout the dermis, but represent a network of functionally distinctive topographic areas (niches) that share one or more common and overlapping molecular mechanisms. Therefore, we performed spatial transcriptomic analysis of 11 biopsies from lesional SSc skin. Detailed methods are available from the corresponding author on request.

Transcriptomes corresponding to 55-μm diameter circular barcodes overlapping vertical skin sections were pooled from all biopsies and analysed with Uniform Manifold Approximation and Projection (UMAP) using previously established bioinformatics pipelines (https://satijalab.org/seurat/articles/spatial_vignette) (Figure 1a). These transcriptomes were then visualized with Loupe Browser (10x Genomics, Pleasanton, CA, USA) (Figure 1b). We identified six distinct transcriptomic clusters, one of which (cluster 2) encompassed the epidermis, while the remaining clusters represented irregular, interwoven zones spanning the entire dermis. Clusters 3 and 5 were associated with hair follicles, and cluster 4 represented pericyte-enriched perivascular areas. Although potentially significant, the small number of barcodes in these clusters limited their interpretability within the scope of our analysis.

**Figure 1:**
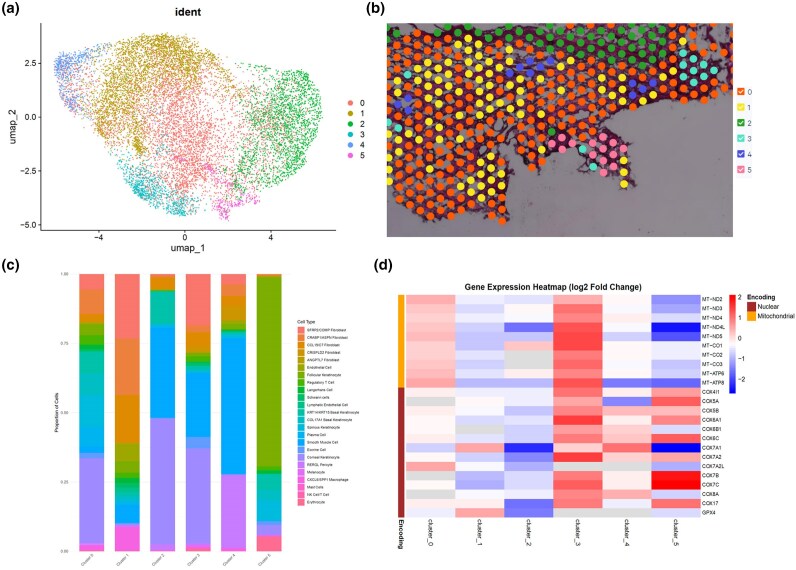
Spatial transcriptomic profile of scleroderma skin. (a) UMAP (Uniform Manifold Approximation and Projection) visualization of SpaceRanger clusters 0–5. (b) Loupe Browser visualization of clustering via SpaceRanger pipeline presenting distinct regions within the histological section of scleroderma skin. (c) Cell type composition of each cluster based on prediction score from single-cell transcriptomic clustering. (d) Heatmap of differential mitochondrial gene expression across six Seurat clusters in systemic sclerosis. Rows represent individual mitochondrial genes, distinguishing nuclear DNA-encoded and mitochondrial DNA-encoded subunits, with clusters (0–5) on the x-axis showing average log2 fold changes. NK, natural killer.

Cell type annotation showed that cluster 1 was predominantly comprised of SSc fibroblast subtypes (*SFRP2*, *CRABP1*/*ASPN*, *CCL19/C7*), complemented by *CXCL9/SPP1* macrophages and smooth muscle cells (Figure 1c).^3^ Query of the differentially expressed genes in each cluster against the Gene Ontology (GO) (geneontology.org and Kyoto Encyclopedia of Genes and Genomes (KEGG) (genome.jp/kegg/) databases confirmed that this cluster represented the primary fibrotic zone (extracellular matrix organization GO:0030198, collagen fibril organization GO:0030199) with a component of an immune response signature (antigen processing and presentation GO:0019882).

Cluster 0 occupied predominantly the upper dermis and encompassed diverse keratinocyte subpopulations along with *CRABP1/ASPN* fibroblasts (Figure 1c), supporting previous observations that *CRABP1/ASPN^+^* fibroblasts may represent fibroblasts found in dermal papillae.^3^ Surprisingly, a prominent fibrotic signature was not observed in this cluster. Instead, its defining characteristic was a strong association with mitochondrial metabolism, marked by an abundance of transcripts linked to the respiratory electron transport chain (GO:0022904) and oxidative phosphorylation (GO:0006119). Additionally, a robust humoral immune response signature (GO:0006959) was evident, though the antigen presentation signature (GO:0019882) observed in cluster 1 was absent.

To further define the mitochondrial changes associated with SSc, we analysed changes in the relative expression levels of mitochondrial DNA (mtDNA)-encoded and nuclear DNA (nDNA)-encoded mitochondrial genes across transcriptomic clusters (Figure 1d). Cluster 0 (and cluster 5) exhibited downregulation of several nDNA-encoded mitochondrial genes, such as *COX4I1*, *COX7C* and *COX6A1*, alongside a compensatory upregulation of mtDNA-encoded mitochondrial genes like *MT-ND4* and *MT-ND5*. This opposing trend between nDNA- and mtDNA-encoded mitochondrial gene expression has been linked with the mitochondrial unfolded protein response (UPR^mt^)^4^ and the integrated stress response (ISR),^5^ and is suggested to be a compensatory mechanism that is activated to maintain mitochondrial function under cellular stress conditions. In contrast, both nDNA-encoded and mtDNA-encoded mitochondrial genes were consistently downregulated in the fibrotic areas (cluster 1), including those encoding key components of aerobic respiration such as *COX4I1*, *COX7C* and *MT-ND4*, indicating total mitochondrial dysfunction and impaired ability to activate UPR^mt^ or engage ISR.

Together, our data using an unbiased, transcriptomic map of SSc skin revealed spatial zonation partitioning the pathogenic inflammatory and fibrotic processes into a zone of inflammation (cluster 0) and fibrosis (cluster 1). Mitochondrial impairment was the most striking difference between these zones and potentially represented an increased mitochondrial stress response in inflammatory zones. Hence, these clusters may represent distinct patterning reflecting sequential temporal phases during the pathogenesis of SSc.

Although the cause of SSc remains unknown, it is likely to be multifactorial. Several environmental and occupational toxins have been linked to an increased risk of SSc, and interestingly, they all share the ability to promote genotoxic stress and toxicity towards mitochondria.^6^ Mitochondrial dysfunction can promote oxidative stress, mutagenesis of nuclear DNA and cellular senescence – all of which are present in SSc.^7,8^ Furthermore, dysfunctional mitochondria may leak DNA into the cytosol, thereby activating the cGAS–STING pathway and instigating type I interferon production, contributing to local inflammation.^9^ Finally, persistent mitochondrial impairment may foster a senescence-associated secretory phenotype,^8^ thereby exacerbating oxidative stress and promoting fibrosis in SSc. We acknowledge that the modest sample size, limited healthy control data and absence of comprehensive functional validation warrant further investigation to fully elucidate these mechanisms.

Although the mechanisms leading to mitochondrial disturbances are undoubtedly heterogeneous and multifactorial, our results indicate that adjunct pharmacological approaches targeting mitochondria may be relevant even in established SSc.

## Data Availability

The raw spatial transcriptomics data generated in this study are available from the corresponding author on request.
